# *Candida auris* Infection in a Meningococcal Septicemia Survivor, Poland

**DOI:** 10.1007/s11046-022-00697-8

**Published:** 2022-12-30

**Authors:** Małgorzata Prażyńska, Patrycja Zalas-Więcek, Tomasz Bogiel, Zbigniew Włodarczyk, Aleksander Deptuła, Marcin Woźniak, Eugenia Gospodarek-Komkowska

**Affiliations:** 1 Department of Microbiology, Ludwik Rydygier Collegium Medicum, Bydgoszcz, Poland; 2 Department of Microbiology, Nicolaus Copernicus University (NCU), Toruń, Poland; 3 Clinical Microbiology Division, Antoni Jurasz University Hospital No. 1, Bydgoszcz, Poland; 4 Department of Transplantation and General Surgery, Ludwik Rydygier Collegium Medicum, Bydgoszcz, Poland; 5 Department of Transplantation and General Surgery, Nicolaus Copernicus University, Toruń, Poland; 6 Department of Transplantation and General Surgery, Antoni Jurasz University Hospital No. 1, Bydgoszcz, Poland; 7 Department of Propaedeutics of Medicine and Infection Prevention, Ludwik Rydygier Collegium Medicum, Bydgoszcz, Poland; 8 Department of Propaedeutics of Medicine and Infection Prevention, Nicolaus Copernicus University, Toruń, Poland; 9 Infection Prevention and Control Team, Antoni Jurasz University Hospital No. 1, Bydgoszcz, Poland; 10 Department of Forensic Medicine, Ludwik Rydygier Collegium Medicum, Bydgoszcz, Poland; 11 Department of Forensic Medicine, Nicolaus Copernicus University, Toruń, Poland

**Keywords:** *Candida auris*, Candidaemia, Infection prevention, MALDI-TOF MS, Poland, Whole genome sequencing

## Abstract

**Background:**

*Candida auris* is an emerging pathogen that constitutes a serious global health threat. It is difficult to identify without specific approaches, and it can be misidentified with standard laboratory methods, what may lead to inappropriate management.

**Case Presentation:**

We report, probably the first in Poland, *C. auris* isolation from blood cultures and wound swabs of a young male following meningococcal septicaemia, in February 2019. The patient had been previously hospitalized in the United Arab Emirates. The isolate was rapidly identified by matrix-assisted laser desorption ionization-time of flight mass spectrometry and therefore clinicians were promptly informed on the alert pathogen isolation. The targeted antifungal treatment was successful and infection control measures seemed effective. ITS-based identification and subsequent whole genome sequencing showed that the *C. auris* isolate belongs to South Asian lineage (clade I).

**Conclusions:**

*C. auris* is able to cause outbreaks in healthcare settings. Therefore, it is important to quickly identify *C. auris* isolates in hospital settings so that healthcare facilities can take proper precautions to limit its spread.

## Background

*Candida auris* has been widely known as an emerging multi-drug resistant fungal pathogen. It might be resistant to all available classes of antifungals [1–4]. This species tends to persist on a human host and on inanimate surfaces for months [5]. In contrast to other *Candida* species, *C. auris* spreads easily in health-care facilities causing sporadic cases and outbreaks [1–5].

Here we report the isolation of *C. auris* from a patient in Poland, the successful targeted antifungal treatment of an invasive infection, and the effective infection control procedures.

## Case presentation

An eighteen years-old male was admitted in February 2019 to the Dr Antoni Jurasz University Hospital No. 1 in Bydgoszcz, Poland, to the Department of Transplantation and General Surgery, for a treatment of severe complications of a meningococcal septicaemia. The patient was transferred directly from the district hospital in Greater Poland, after two days of hospitalization and following three weeks of hospitalization in the United Arab Emirates (UAE), due to a meningococcal septicaemia.

**Fig. 1:**
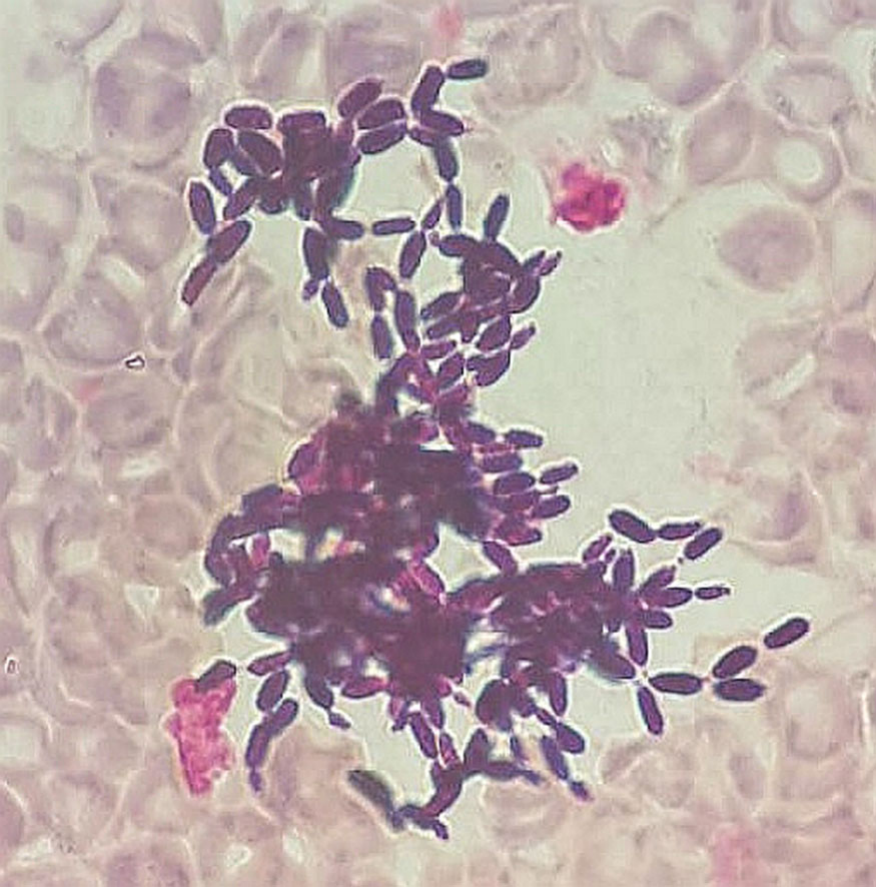
Microscopic examination of positive blood sample with *C. auris*

At the admission, two blood samples and seven swabs from necrotic chronic wounds were collected. The aerobic blood culture became positive after two days of incubation (BD BACTEC™ FX system, Becton, Dickinson and Company, Sparks, MD, USA). Microscopic examination revealed the presence of budding, non-pseudohyphae-forming, cylindrical shaped yeast cells (Fig. 1). An empiric treatment with an intravenous fluconazole was administered.

### Identification of *Candida auris*

Matrix-assisted laser desorption ionization-time of flight mass spectrometry (MALDI-TOF MS) using the MALDI Biotyper system (Bruker Daltonics, Bremen,Germany) provided a confident species-level identification of the isolate (score 2.16). The clinicians were immediately informed on *C. auris* isolation.

Besides, multifocal *C. auris* colonization of necrotic chronic wounds on upper and lower limbs, and subsequently—stumps, was documented. A total of 20 *C. auris* isolates were cultured (Table 1).

**Table 1:** *C. auris* isolations

Date of isolation	Specimen	Susceptibility
13-Feb-2019	Blood (aerobic culture)	Fluconazole: > 256 mg/L Amphotericin B: 1 mg/L anidulafungin: 0.047 mg/L Caspofungin: 0.25 mg/L Micafungin: 0.064 mg/L Voriconazole: 0.25 mg/L
13-Feb-2019	Chronic wound swab (right thigh)	Fluconazole: > 256 mg/L Amphotericin B: 1.5 mg/L Anidulafungin: 0.047 mg/L Caspofungin: 0.25 mg/L Micafungin: 0.064 mg/L Voriconazole: 0.25 mg/L
13-Feb-2019	Chronic wound swab (right crus)	Micafungin: 0.064 mg/L
13-Feb-2019	Chronic wound swab (left crus)	Micafungin: 0.064 mg/L
13-Feb-2019	Chronic wound swab (right foot)	Micafungin: 0.064 mg/L
13-Feb-2019	Chronic wound swab (right palm)	Micafungin: 0.064 mg/L
13-Feb-2019	Chronic wound swab (left palm)	Micafungin: 0.064 mg/L
13-Feb-2019	Chronic wound swab (left forearm)	Micafungin: 0.064 mg/L
14-Feb-2019	Catheter-drawn blood (aerobic culture)	Fluconazole: > 256 mg/L Amphotericin B: 1 mg/L Anidulafungin: 0.047 mg/L Caspofungin: 0.25 mg/L Micafungin: 0.064 mg/L
17-Feb-2019	Catheter-drawn blood (aerobic culture)	Amphotericin B: 1.5 mg/L Micafungin: 0.125 mg/L
17-Feb-2019	Catheter-drawn blood (new catheter, aerobic culture)	Amphotericin B: 1.5 mg/L Micafungin: 0.125 mg/L
20-Feb-2019	Chronic wound swab (right crus)	Micafungin: 0.125 mg/L
20-Feb-2019	Chronic wound swab (left crus)	Micafungin: 0.125 mg/L
27-Feb-2019	Wound swab (stump)	Micafungin: 0.125 mg/L
27-Feb-2019	Catheterized urine sample (single colonies)	Testing not performed
07-Mar-2019	Wound swab (right leg stump)	Micafungin: 0.125 mg/L
07-Mar-2019	Wound swab (left leg stump)	Micafungin: 0.125 mg/L
12-Mar-2019	Wound swab (right leg stump)	Micafungin: 0.125 mg/L
20-Mar-2019	Chronic wound swab (left buttock)	Micafungin: 0.125 mg/L
26-Mar-2019	Wound swab (right leg stump)	Micafungin: 0.125 mg/L

### Antifungal Susceptibility Testing

Antifungal susceptibility testing was performed by Etest method (bioMérieux, Marcy-l'Étoile, France) according to the manufacturer's instructions. The following minimum inhibitory concentrations (MICs) values were observed for the first obtained isolate: fluconazole > 256 mg/L, amphotericin B 1 mg/L, anidulafungin 0.047 mg/L, caspofungin 0.25 mg/L, micafungin 0.064 mg/L. The results of the susceptibility tests performed on subsequently obtained *C. auris* isolates are presented in Table 1. The results interpretation was performed according to CDC guidelines (Centers for Disease Control and Prevention, CDC) [1].

### Treatment

Therapy with micafungin 100 mg/day was initiated. Octenilin^®^ and Octenisept^®^ (Schülke & Mayer, Norderstedt, Germany) were used topically. Altogether, four positive blood cultures were obtained, all in aerobic conditions. Simultaneously incubated blood cultures in an anaerobic conditions were negative. The first negative blood culture in the follow-up investigation was noticed after seven days of treatment with micafungin. The treatment was continued for two weeks in total. The susceptibility of the isolates to micafungin was monitored, and they remained susceptible to the drug. However, a MIC value increase from 0.047 to 0.125 mg/L was observed.

In contrast to the eradication of *C. auris* from blood, a persistent colonization of necrotic chronic wounds of upper and lower limbs with *C. auris* was documented.

### Infection Prevention and Control

According to the local procedures, contact precautions were implemented from the admission day, but the ICU and Surgical Department staff were additionally warned about the epidemic potential of *C. auris*. Any additional changes for environmental cleaning methods were not applied, since regularly used disinfectants in our hospital have sufficient antifungal activity—Incidin Plus (active ingredient (AI): glucoprotamin), Incidin Pro (AIs: 2-phenoxyethanol, N,N-bis-(3-aminopropyl) dodecylamine, benzalkoniumchloride), Incidin Liquid Spray (AIs: 2-propanol, 1-propanol, amphoteric surfactants), Sani Cloth Active (AI: didecyldimethylammonium chloride), and Incides N (AIs: 2-propanol, 1-propanol) (Ecolab, Kraków, Polska).

### Molecular Characterization

#### Culture for DNA Isolation

DNA was isolated from the 48 h culture of the clinical *C. auris* isolate (no. 5049–2019) and type *C. auris* DSMZ 21,092 strain with an application of ExtractMe DNA Yeast Kit (DNA Gdańsk, Gdańsk, Poland), performed according to manufacturer’s instruction. The DNA samples concentration quality was checked spectrophotometrically and the isolation protocol efficiency was confirmed additionally electrophoretically in 1.5% agarose in TBE buffer (Bio-Rad, Feldkirchen, Germany). DNA quality control was performed by measuring the absorbance at 260/280 nm, the template concentration was determined using Qubit fluorimeter (Thermo Fisher Scientific, Waltham, Massachusetts, USA). DNA integrity was analyzed by 0.8% agarose gel electrophoresis.

#### ITS Region Sequencing

The ribosomal DNA ITS-region was amplified using primers ITS1 and ITS4 as previously described [6]. An amplification was performed using an Applied Biosystems 9700 Thermal Cycler according to cycling conditions described by Carolus et al. [6]. An amplification results were checked on a 1% agarose in TAE buffer. PCR product was cleaned using Amicon Ultra 100 kDa filters (Millipore, Burlington, Massachusetts, USA) and sequenced using BigDye™ Terminator v3.1 Cycle Sequencing Kit (Thermo Fisher Scientific) according to manufacturer's instructions. The sequencing products were electrophoresed on an ABI 3130 DNA analyzer using 36 cm capillaries and POP-7 polymer. The sequences were compared with these corresponding to a particular clade [6]. The type *C. auris* DSMZ 21,092 strain DNA sequence of the ITS locus was identical to the reference sequence of clade II. Clinical *C. auris* isolate was recognized as clade I or III, therefore whole genome sequencing (WGS) was performed to define the clade.

#### Whole Genome Sequencing

Paired-end sequencing library was constructed using the NEB Ultra II FS Preparation Kit (New England Biolabs, Beverly, USA) according to the manufacturer’s instructions. Library was sequenced using an Illumina NextSeq platform (Illumina, San Diego, USA) with 2 × 75 paired-end reads. Sequence quality metrics were assessed using FASTQC (http://www.bioinformatics.babraham.ac.uk/projects/fastqc/) [7] and quality trimmed using fastp [8].

### Genome Assembling and Phylogenetic Analysis

Filtered Illumina reads were assembled into contigs using SPAdes v.3.13.1 [9]. Phylogenetic analysis was performed using mycosnp pipeline v1.3 (https://github.com/CDCgov/mycosnp-nf) [10] with default parameters.

Illumina sequencing yielded 131 million reads and 9.88 Gb of sequence data. Genome assembly generated 930 contigs with N50 41 kb and total assembly size was 12,257,419 bp. Clade identification strategy was done as described previously [11]. In brief, seven additional genomes of *C. auris* from National Center for Biotechnology Information (NCBI) BioProject PRJNA328792 [12] were included in the analysis to help with assignment of clades I-IV; clade I: B11112 (SAMN05379584), B11201 (SAMN05379593); clade II: B11220 (SAMN05379608); clade III: B11221 (SAMN05379609), B11230 (SAMN05379618); clade IV: B11852 (SAMN09111946), B11243 (SAMN05379619). The here generated whole genome sequences of *Candida*-*auris*-PL1 isolate have been deposited in NCBI Genome under accession numbers: BioProject: PRJNA863457, SRA: SRS1478113, BioSample: SAMN30096895.

A genomic relationship of tested *C. auris* isolate (*Candida-auris*-PL1) reveals that it belongs to clade I. The reference strain for the phylogeny analysis was *C. auris* B8441, which belongs to clade I (NCBI Genbank ID: PEKT00000000.2). WGS was performed and the obtained data were analysed at the DNA Sequencing and Oligonucleotide Synthesis Laboratory, Institute of Biochemistry and Biophysics of the Polish Academy of Sciences (IBB PAS).

## Discussion and Conclusions

*C. auris* was firstly identified in 2009, when isolated from the external ear canal of an inpatient in a Japanese hospital [13]. Next, within a few years, *C. auris* has emerged as healthcare-associated alert pathogen. *C. auris* isolations, sporadic cases and outbreaks, have been reported in around 40 countries, on six continents: Asia (2011, retrospective study—1996), Africa (2014), Europe (2015), South America (2016), North America (2016), and Australia (2018) [1–4].

WGS analysis of clinical *C. auris* isolates discovered four major clades: South Asian (clade I), East Asian (clade II), African (clade III), South American (clade IV), and Iranian (clade V), which means simultaneous emergence of multidrug-resistant *C. auris* on different continents [12, 14, 15]. Simultaneously, Tsay et al. [16] revealed the transmission of *C. auris* isolates in health care facilities within several United States.

*C. auris* strains firstly appeared in Europe in 2015 [5]. The outbreak involved over 50 cases in a London hospital (2015–2016), and the ward closure was implemented to control outbreak. Thereafter, sporadic cases and outbreaks in Europe have been reported from: Austria, Belgium, France, Germany, Greece, Italy, the Netherlands, Norway, Spain, Switzerland, and as we here reported, in Poland [3, 4, 17–20].

To our knowledge this case is the first report of *C. auris* from Poland. Applied molecular methods confirmed that the isolate belongs to clade I, also known as South Asian. It is presumed that *C. auris* infection and colonization was ongoing when the patient was admitted to our hospital, but the *Candida* sp. was not isolated or identified to the species-level in the United Arab Emirates (UAE). A sporadic case of *C. auris* isolation in the UAE has been previously reported [21]. Widely used phenotypic methods mostly fail to identify this novel *Candida* species. At present, mass spectrometry (e.g. MALDI Biotyper, Bruker, and Vitek MS, bioMérieux) and DNA sequencing of the ITS and/or D1/D2 ribosomal DNA regions reliably identify *C. auris* [1, 22]. Still there is inability to identify *C. auris* accurately in a large number of countries.

Fortunately, we were able to identify the pathogen rapidly and reliably, and inform the clinician promptly. Hence, contact precautions were introduced immediately to prevent this pathogen transmission.

The actual problem of *C. auris* is a lack of standards for disinfection procedures effective against *C. auris*. The protocols applied for infection control in our healthcare facility seem effective as until time of publication, any other *C. auris* isolation ensued.

Likewise the majority of *C. auris* strains [1, 3], our isolate was resistant to fluconazole, therefore the treatment with echinocandin was required. We have monitored the antifungal susceptibility of the isolates during therapy, and they remained susceptible to echinocandins until the end of the treatment.

*C. auris* tends to persists in patients, especially on skin of groins and axillae, in ear canals, and in nostrils [5]. We have also observed persistent colonization of the chronic wounds on limbs, over a month after a successful treatment of candidaemia.

Our case proved that careful and precise *Candida* spp. identification to the species level is of great importance. There is a high need of a reliable and rapid identification of *C. auris* in all medical microbiology laboratories using new technologies approved for microbiological diagnostic. This is the basic tool for an effective prevention of infection and control measures.

In conclusion, our report confirms the ongoing introduction of *C. auris* into European hospitals and the role of a reliable and rapid *C. auris* identification in prevention of further transmission and healthcare-associated outbreaks. Difficulties in an identification may delay early pathogen detection, increasing the risk for *C. auris* transmission. A cooperation of microbiologists, clinicians, nurses and epidemiologists is particularly important in this case. There is a constant need to evolve standards for infection prevention and control measures effective against *C. auris*.

## Data Availability

DNA sequencing data are available in NCBI: BioProject: PRJNA863457, SRA: SRS14781133, BioSample: SAMN30096895.
